# Functional connectivity of music-induced analgesia in fibromyalgia

**DOI:** 10.1038/s41598-019-51990-4

**Published:** 2019-10-29

**Authors:** Victor Pando-Naude, Fernando A. Barrios, Sarael Alcauter, Erick H. Pasaye, Lene Vase, Elvira Brattico, Peter Vuust, Eduardo A. Garza-Villarreal

**Affiliations:** 1 Subdirección de Investigaciones Clínicas, Instituto Nacional de Psiquiatría “Ramón de la Fuente Muñiz”, México City, México; 2 Institute of Neurobiology, Universidad Nacional Autónoma de México campus Juriquilla, Querétaro, México; 3 Center for Music in the Brain, University of Aarhus, Aarhus, Denmark; 4 Department of Behavioral and Cognitive Neurobiology, Brain Mapping Lab, Institute of Neurobiology, Universidad Nacional Autónoma de México campus Juriquilla, Querétaro, México; 5 Magnetic Resonance Unit, Institute of Neurobiology, Universidad Nacional Autónoma de México campus Juriquilla, Querétaro, México; 6 Department of Psychology and Behavioral Sciences, University of Aarhus, Aarhus, Denmark; 7 Danish Pain Research Center, Aarhus University Hospital, Aarhus, Denmark; 8 Royal Academy of Music, Aarhus, Denmark; 9 Laboratorio Nacional de Imagenología por Resonancia Magnética (LANIREM), Institute of Neurobiology, Universidad Nacional Autonoma de Mexico (UNAM) campus Juriquilla, Queretaro, Mexico

**Keywords:** Chronic pain, Fibromyalgia

## Abstract

Listening to self-chosen, pleasant and relaxing music reduces pain in fibromyalgia (FM), a chronic centralized pain condition. However, the neural correlates of this effect are fairly unknown. In our study, we wished to investigate the neural correlates of music-induced analgesia (MIA) in FM patients. To do this, we studied 20 FM patients and 20 matched healthy controls (HC) acquiring rs-fMRI with a 3T MRI scanner, and pain data before and after two 5-min auditory conditions: music and noise. We performed resting state functional connectivity (rs-FC) seed-based correlation analyses (SCA) using pain and analgesia-related ROIs to determine the effects before and after the music intervention in FM and HC, and its correlation with pain reports. We found significant differences in baseline rs-FC between FM and HC. Both groups showed changes in rs-FC after the music condition. FM patients reported MIA that was significantly correlated with rs-FC decrease between the angular gyrus, posterior cingulate cortex and precuneus, and rs-FC increase between amygdala and middle frontal gyrus. These areas are related to autobiographical and limbic processes, and auditory attention, suggesting MIA may arise as a consequence of top-down modulation, probably originated by distraction, relaxation, positive emotion, or a combination of these mechanisms.

## Introduction

Music-induced analgesia (MIA) is defined as the subjective reduction of pain perception after listening to music^1^, and the effect has been reported in chronic pain conditions such as low back pain, osteoarthritis, and fibromyalgia^2–4^. Endogenous pain inhibition depends on the descending pain modulatory system (DPMS), with areas involved such as the dorsolateral prefrontal cortex (dlPFC), periaqueductal gray matter (PAG) and rostral ventral medulla (RVM)^5–7^. Thus, the possible neural mechanisms of MIA are suggested to be top-down through the DPMS, secondary to cognitive and emotional mechanisms such as distraction^8,9^, familiarity^10,11^, emotion^12^, relaxation and reward^13,14^. MIA may be then catalogued as a centralized type of analgesia, given that the effect seems to originate in the brain and not by peripheral nociceptive receptors^15^.

Fibromyalgia (FM) is a chronic pain syndrome of unknown etiology that predominantly affects women, and is characterized by increased sensitivity to somatosensory nociception, and associated with other symptoms such as sleep disorders, stiffness, fatigue, anxiety, and depression^16–20^. There is still no specific treatment for FM and conventional treatment can result in abuse of painkillers, which lead to other co-morbidities^21^. FM patients seem to exhibit a decrease of central inhibition or facilitation of the nociceptive input in the DPMS^22–24^, and thus are more sensitive to pain, as well as other types of sensory input such as noise^25^. This seems to be a consequence of increased function of the pain pathways, increased membrane excitability and synaptic efficacy, as well as reduced neuronal inhibition^26^.

In FM, studies have investigated morphological and functional characteristics of these patients using different neuroimaging techniques^27^. Specifically, recent resting-state fMRI studies have found alterations in brain connectivity in FM patients^28–31^, involving networks related to pain intensity and analgesia^18,32–34^. FM patients have shown increased resting state functional connectivity (rs-FC) of areas related to pain processing, and reduced connectivity in regions involved in pain inhibitory modulation^35^. These findings include connectivity changes of areas such as insula, thalamus, amygdala, PAG, anterior and posterior cingulate cortex, medial prefrontal cortex, primary motor cortex, primary and secondary somatosensory cortices, basal ganglia, and prefrontal areas^18,30,35–39^. Therefore, alterations in rs-FC in FM patients appear to involve not only areas related to pain processing (perception and modulation), but also related to somatomotor, executive, limbic, autobiographic, and integration processes.

In terms of MIA, there are no existing neuroimaging studies in acute or chronic pain, with the exception of our own previous study, where we showed MIA related to increased BOLD signal amplitude in the angular gyrus (AnG) in FM patients^40^. Although the changes in amplitude were correlated with the pain self-report, the study did not include healthy controls to contrast changes in functional connectivity. Overall, if the mechanisms behind MIA are related to the DPMS, areas such as the ACC, PAG and INS should show rs-FC changes in FM patients. In this study, we wished to investigate rs-FC patterns of MIA in FM patients, compared to age and sex-matched healthy controls (HC), by means of pain self-report, rs-fMRI and seed-based correlation analyses on the rs-FC of a customized pain matrix. We hypothesized that (1) FM patients would show significant differences in rs-FC of areas related to pain processing at rest, and (2) the analgesic effect of music would be associated to changes in rs-FC of areas related to the DPMS in FM patients.

## Materials and Methods

### Participants

The study was conducted at the Instituto de Neurobiología of the Universidad Nacional Autónoma de México (UNAM Juriquilla, Queretaro, Mexico). A fibromyalgia group (FM, n = 20, age range = 22–70, mean = 46.4, SD = 12.4) and an age-matched control group (HC, n = 20, age range = 21–70, mean = 42.1, SD = 12.5) participated in this study. Given the difficulties of obtaining male participants with a complete diagnose of FM, and the fact that it affects predominantly women, our entire sample included only women. The inclusion and exclusion criteria for participation in the fMRI experiment is described in Table 1 ^17,20^.

**Table 1:** Participant selection criteria.

**Inclusion Criteria for Fibromyalgia Patients**.
• Meeting the Fibromyalgia1990 and 2010 criteria.
• Woman with Fibromyalgia diagnosed by a trained Rheumatologist.
• Spontaneous, continuous and intense pain in daily life (VRS > 5 average of a month)
• Right-handed.
**Inclusion Criteria for Healthy Controls**.
• Healthy Adult Woman.
• Right-handed.
**Exclusion Criteria for Fibromyalgia Patients and Healthy Controls**.
• Impossibility to move or walk.
• Uncontrolled endocrine problems.
• Neurological alterations (i.e. stroke, epilepsy, recent traumatic brain injury).
• Auditory problems.
• MRI contraindications (i.e., metal prosthetics).
• Pregnancy and/or breast-feeding.
**Elimination Criteria for Fibromyalgia Patients and Healthy Controls**.
• Excessive artifacts in MRI.
• Probable pathological findings in MRI.

VRS, Verbal Rating Scale.

All participants gave their consent verbally and in written form before the experiment. FM patients were asked not to intake painkillers at the day of testing only. The HC group were screened to ensure that they did not experience any type pain at the day of testing. The study was conducted in accordance with the Declaration of Helsinki and approved by Bioethics Committee of the Instituto de Neurobiología, UNAM. Patients received no compensation for participating in the study.

### Design and paradigm

Part of the current data has been previously analyzed and published by Garza-Villarreal *et al*.^40^, which showed that MIA related to increased BOLD signal amplitude in the angular gyrus (AnG) in FM patients by analyzing fractional amplitude of low frequency fluctuations (fALFF). This method obtains differences in BOLD signal amplitude between conditions. In this new study, we included healthy controls to determine if the effect of music on the brain is in fact related to analgesia, and performed seed-based functional connectivity analyses of a pain-related matrix. Participants answered the Pain Catastrophizing Scale (PCS)^41^, the State-Trait Anxiety Inventory (STAI)^42^, the Pain Self-Perception Scale (PSP)^43^, and the Center for Epidemiologic Studies Depression Scale (CES-D)^44^ before the MRI scanning, to establish clinical and behavioral differences between FM patients and HC. It must be noted that these scales were not used for diagnosis, only used to measure symptoms.

**Figure 1:**
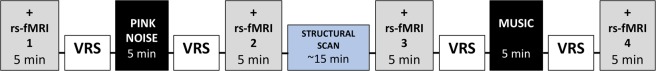
Experimental rs-fMRI Paradigm. Experimental conditions were pink noise and music. Image acquisitions were performed before and after each experimental condition, in which participants were instructed to stay alert with eyes opened and fixated on a white cross displayed on the center of a black background presented on a screen. Pain Intensity and Pain Unpleasantness was reported by fibromyalgia patients only, before and after each experimental condition. The washout condition was executed during the “Structural Scan” period. PINK NOISE, control condition; VRS, pain verbal rating scale; rs-fMRI, resting state functional magnetic resonance imaging; +, fixation cross.

To evaluate pain while in the MRI scanner, pain intensity (PI) and pain unpleasantness (PU) were measured only in FM patients (Fig. 1), using the verbal rating scale (VRS) (0 = no pain, 10 = worst pain possible)^45^. PI refers to the sensory aspect of pain, whereas PU refers to the evaluative and emotional dimension of pain^46^. PI and PU were measured immediately before and after each experimental condition. The experimental conditions consisted of five-minute long auditory tracks, either music or pink noise (control), presented while no imaging was acquired, with participants inside the MRI scanner. Prior to the study, participants provided a list of songs or artists that they would like to listen during the experiment. Songs had to be familiar, highly pleasant and slow paced. The slow pace was defined as a tempo of <120 beats per minute (bpm), determined by the researcher using a metronome. Pleasantness was reported by the participant using a 10-point verbal scale (0 = unpleasant, 10 = highly pleasant), and to be selected, the song had to be rated at least 9–10 points. When only the artist name was provided, the researcher chose the songs based on two fixed acoustic criteria: consonance (pleasantness), verbally reported by the participant, and slow tempo. We conducted a previous systematic review and meta-analysis^47^ of publications that reported music-induced analgesia in chronic pain conditions, and we found a higher effect when participants choose familiar music to listen, making self-choice and familiarity important mechanisms of this effect. Pink noise was selected by a prior pilot study in which several types of noise were presented to healthy participants^48^, and pleasantness ratings were obtained. Pink noise resulted as more neutral than other types of noise (i.e. white noise).

Participants listened to the auditory stimuli inside the MRI scanner (Fig. 1), a period in which no sequences were acquired to minimize unwanted noise. The order of the auditory stimulus presentation was counter-balanced across participants, to avoid any order effect. Auditory stimuli were presented using the NordicNeuroLab AS (Bergen, Norway) MRI-safe headphones. For each session, there were a total of four rs-fMRI acquisitions (rs-fMRI 1, rs-fMRI 2, rs-fMRI 3, rs-fMRI 4) lasting five minutes each, in which participants were instructed to stay alert with eyes opened and not to fixate on a cross presented on the screen. A wash-out condition was presented between the second and third functional acquisition, and consisted of watching a video documentary with sound (i.e., a biography of Bill Gates), period in which structural imaging was acquired (Fig. 1). The purpose of the wash-out condition was to avoid analgesic or cognitive cross-over effects. Visual stimuli (fixation and wash-out condition) were presented in a screen projected through a mirror mounted on the MRI head coil, using the software VLC Media Player (http://videolan.org). A total of eight conditions were defined for the statistical analysis: pre-control (Cpre), post-control (Cpos), pre-music (Mpre), and post-music (Mpos) for both groups (FM and HC). A baseline (BL) condition was defined as the first rs-fMRI sequence acquired by participant, and it was used to analyze differences in brain functional connectivity between groups (FM and HC) before the music intervention, and to select relevant brain areas to analyze the neural correlates of MIA.

### Procedure

FM patients were recruited through a fibromyalgia support group and from the General Hospital of the Health Government Department (Hospital General de la Secretaría de Salud) both located in Queretaro, Mexico. HC were recruited using flyers placed in the Instituto de Neurobiología, and with the help of students and workers of the same institute. Potential participants were informed and interviewed by phone to make sure they met the inclusion criteria. After participants were confirmed to be eligible and accepted to participate in the study, they were asked for songs they would like to listen to during the study, that would fit the characteristics described in the previous section. Before the MRI scans, they were briefed about the study to make sure they understood the procedure and implications. Participants then answered the behavioral questionnaires described above. During the MRI scanning, participants in the FM group rated their spontaneous pain immediately before and after each auditory condition. The HC group experienced no pain; thus, pain was not measured.

### MRI data acquisition

The image acquisition was performed with a 3.0 Tesla GE Discovery MR750 scanner (HD, General Electric Healthcare, Waukesha, WI, USA) and a commercial 32-channel head coil array. High-resolution T1-weighted anatomical images were obtained using the FSPGR BRAVO pulse sequence: plane orientation = sagittal, TR = 7.7 ms, TE = 3.2 ms, flip angle = 12°, matrix = 256 × 256, FOV = 256 mm^2^, slice thickness = 1 mm, number of slices = 168, gap = 0 mm, slice order = interleaved, view order = bottom-up. A gradient echo sequence was used to collect rs-fMRI data using the following parameters: plane orientation = axial, TR = 3000 ms, TE = 40 ms, flip angle = 90°, matrix = 128 × 128, FOV = 256 mm^2^, slice thickness = 3 mm, voxel size = 2 × 2 mm, number of slices = 43, gap = 0 mm, slice order = interleaved, view order = bottom-up. The total time for each rs-fMRI sequence was 5 minutes with a total of 100 brain volumes acquired per run, with 4 runs per subject. During the scanning, patients were not given any instructions about the music or pink noise, but were instructed to stay alert, to keep their eyes open without thinking anything in particular. All images were saved in DICOM format, anonymized and converted to NIFTI format using dcm2nii from MRIcron^49^.

### Statistical analysis of questionnaires and pain measures

Descriptive and inferential statistics of data and plots were performed using R software^50^ and the “ggplot2” package of R^51^. To establish behavioral differences between experimental groups (FM and HC), a two-tailed unpaired *t-student* test was performed on the results from pain self-perception, pain catastrophizing, anxiety, and depression questionnaires. PI and PU scores of the FM group were not normally distributed, therefore, non-parametric two-tailed paired analyses were performed with the *Mann-Whitney Rank* test. This analysis was performed in the difference of variables ΔPI (pre-post PI) and ΔPU (pre-post PU) between the two experimental conditions (music and pink noise).

### Functional connectivity analysis

The rs-fMRI data was preprocessed and analyzed using the CONN Toolbox for Matlab^52^. Structural and functional images were imported into CONN and the preprocessing pipeline included: realignment, slice-timing correction, structural segmentation and spatial normalization (simultaneous Gray/White/CSF segmentation and normalization to the MNI space), outlier detection (ART-based identification of outlier scans for scrubbing; subject motion correction = 2.5 mm and global signal Z-value threshold = 3), and smoothing (spatial convolution with a Gaussian kernel with FWHM = 5 mm). Nuisance variables were regressed out using the general lineal model. Signal timeseries were band-pass filtered between 0.008 and 0.09 Hz. Nuisance variables included six motion variables, and principal components of white matter and cerebrospinal fluid, a method referred as aCompCor^53^. The aCompCor avoids artefactual anticorrelations introduced by global signal regression and reduces artifact by physiological signals. To evaluate resting state functional connectivity (rs-FC) we performed seed-based correlation analysis (SCA). This analysis measures the correlation of the time-series of the BOLD signal (blood oxygen level dependent signal) of a specific region-of-interest (ROI) with the rest of the whole brain (seed-to-voxel approach).

**Figure 2:**
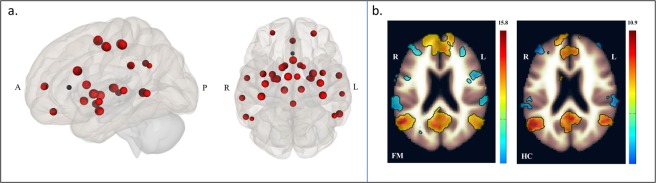
Pain matrix rs-fMRI seed-based correlation analysis. (**a**) 3D image of the pain matrix: ACC, anterior cingulate cortex; PCC, posterior cingulate cortex; BA41, primary auditory cortex; AMYG, amygdala; AnG, angular gyrus; CAU, caudate; GP, globus pallidus; PUT, putamen; INS, insular cortex; mPFC, medial prefrontal cortex; PAG, periaqueductal gray matter; M1, primary motor cortex; SI, primary somatosensory cortex; SII, secondary somatosensory cortex; SMA, supplementary motor area; STS, superior temporal sulcus; THA, thalamus. (**b**) Connectivity maps of the pain matrix for FM (left) and HC (right) respectively. We calculated the BOLD mean time-series of all 34 seeds into one single *a priori* brain mask, and correlated it with the rest of the whole brain. Colors show either positive (red–yellow) or negative (blue–light blue) correlations of the pain matrix. Image shows positive correlations of the pain matrix with the Default Mode Network (DMN), with areas such as mPFC, PCC, PCN, and AnG.

We divided the analysis into four consecutive steps. For step-1 or “seed definition”, our seeds were created from 34 ROIs using fslmaths^54^ with a 5-mm kernel sphere, each in MNI space. The ROIs were obtained from peak coordinates of several pain studies, both experimental and clinical^28,35,40,55–57^, and cross-referenced with brain atlases (Harvard-Oxford atlas FSLview; Juelich Histological Atlas FSLview), and Neurosynth^58^ using “pain” and “chronic pain” as search terms, to ensure the precision of the obtained coordinates. Most of the seeds were derived from the study by Cifre *et al*.^35^, given that it showed for the first time a disrupted brain functional connectivity in FM patients. The seeds were located on the following brain regions on both hemispheres: anterior cingulate cortex (ACC), angular gyrus (AnG), amygdala (AMYG), primary auditory cortex (BA41), caudate (CAU), globus pallidus (GP), putamen (PUT), insula (INS), medial prefrontal cortex (mPFC), periaqueductal grey matter (PAG), posterior cingulate cortex (PCC), primary motor cortex (M1), primary somatosensory cortex (SI), secondary somatosensory cortex (SII), supplementary motor area (SMA), superior temporal sulcus (STS), and thalamus (THA) (Fig. 2, Supplementary Table 1). Furthermore, we merged all 34 ROIs together to create an *a priori* brain mask using fslmerge^54^, which concatenated all seeds into one single output. Because most of these regions, but not all, are part of the so-called pain network, we named this merged seed “pain matrix”. We performed seed-to-voxel whole-brain correlation analyses for both modalities: (1) the pain matrix and (2) by each seed independently. All rs-FC analyses were corrected for multiple comparisons using the false discovery rate (FDR) at q = 0.05.

For step-2 or “baseline rs-FC contrast”, we compared the resulting correlation maps of only the baseline (BL) condition between groups (FM vs HC) using a general linear model (GLM, α = 0.05) between-subjects design with the following covariates: age, years with FM diagnosis, and anxiety and depression symptoms. The rs-fMRI data for this analysis was taken from the participant’s Cpre and Mpre conditions (before music, and before pink noise). The purpose of this was to identify specific areas already affected in FM that may change in relation to MIA. The seeds with significant differences in rs-FC between groups were included in step-3.

For step-3 or “rs-FC of MIA”, we investigated the main effects of group (FM & HC), condition (music & pink noise) and time (pre- & post-test), and a 3-way interaction on rs-FC changes of the pain matrix. We conducted an GLM (α = 0.05), on a *post hoc* pain matrix that resulted from the step-2 analysis (BL). In a 2 × 2 × 2 ANOVA, we modeled the different conditions (8 conditions; FM_Cpre, FM_Cpos, FM_Mpre, FM_Mpos, HC_Cpre, HC_Cpos, HC_Mpre and HC_Mpos) and conducted 2-sided F-statistics to test main effects and interactions. Then, we conducted *post hoc* paired t-tests within each group to assess specific rs-FC changes from pre to post-conditions.

For step-4 or “correlation of rs-FC and MIA”, we determined if the analgesic effect of music correlated with rs-FC results in FM patients using ΔPI and ΔPU scores, along with the rs-FC contrast results of Mpre vs Mpos (Δrs-FC) values. The purpose of this analysis is to correlate subjective data (pain self-report) with objective data (rs-fMRI). All scores were transformed to Z-values and a two-tailed Pearson´s correlation analysis was performed, considering it significant at α = 0.05.

### Statement of significance

This original article explores the neural correlates of music-induced analgesia in a prototypical chronic pain disorder such as fibromyalgia. Using state-of-the-art neuroimaging techniques, we show that music reduces pain, and that the effect correlates with changes in brain functional connectivity of areas related to pain and analgesia.

## Results

### Questionnaires and pain measures

As expected and in accordance with previous FM behavioral studies, pain self-perception, pain catastrophizing, anxiety and depression were significantly different between FM and HC (Table 2). The t-student test showed that patients with FM report greater pain self-perception (<0.001), pain catastrophizing (p < 0.001), anxiety (p < 0.001) and depression (p < 0.001) symptoms than HC. The Mann-Whitney Rank test showed that pink noise and music were significantly different in both ΔPI (W = 60, p = 0.002) and ΔPU (W = 65.5, p = 0.004). In other words, FM patients reported lower pain levels after listening to music, but not after listening to the pink noise. Pink noise produced a small increase of pain perception in FM patients (~1 point VRS), which was non- significant in both PI and PU (Supplementary Fig. 1).

**Table 2:** Descriptive and Inferential Statistics of Behavioral Questionnaires.

Group	FM (n = 20)	HC (n = 20)	p
	Mean ± SD	Mean ± SD	
Age	46.4 ± 12.4	42.1 ± 12.5	0.28
PCS	27.6 ± 12.5	12 ± 10.9	<**0**.**001**
• Helplessness	13.3 ± 5.7	4.9 ± 4.9	<**0**.**001**
• Magnification	5.3 ± 3.8	2.6 ± 2.6	**0**.**012**
• Rumination	9.3 ± 4.6	4.5 ± 4.2	**0**.**001**
PSP	56.1 ± 28.7	17.6 ± 23.7	<**0**.**001**
STAI	52.8 ± 20.1	26.1 ± 10.7	<**0**.**001**
• State	19.5 ± 10.6	11.8 ± 7.1	**0**.**01**
• Trait	33.3 ± 12.8	14.3 ± 5.8	<**0**.**001**
CES-D	31 ± 13.7	11 ± 8.6	<**0**.**001**

FM, fibromyalgia; HC, healthy controls; PCS, pain catastrophizing scale; PSP, pain self-perception scale; STAI, state-trait anxiety inventory; CES-D, center for epidemiologic studies depression scale.

### Functional connectivity

#### Baseline contrast: FM patients display altered rs-FC of the pain matrix

**Figure 3:**
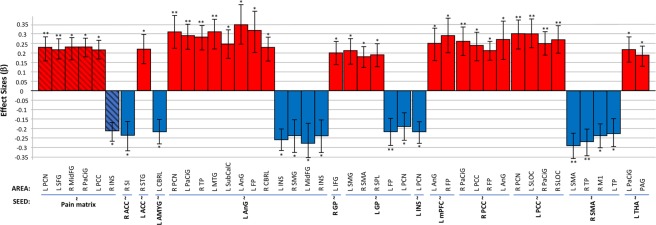
Baseline Condition FM vs HC. Significant seed-to-voxel rs-FC of the pain matrix; positive effects represent higher connectivity; negative effects represent lower connectivity. FM, fibromyalgia; HC, healthy controls; L, left; R, right. **Significant Seeds**: Pain matrix (*34 seeds merged in an *a priori* mask); ACC, anterior cingulate cortex; AMYG, amygdala; AnG, angular gyrus; GP, globus pallidus; INS, insular cortex; mPFC, medial pre-frontal cortex; PCC, posterior cingulate cortex; SMA, supplementary motor area; THA, thalamus. **Correlated areas**: AnG, angular gyrus; CRBL, cerebellum; FP, frontal pole; IFG, inferior frontal gyrus; INS, insular cortex; SLOC, superior lateral occipital cortex; MTG, medial temporal gyrus; MidFG, middle frontal gyrus; PaCiG, paracingular gyrus; PAG, periaqueductal gray matter; M1, precentral gyrus (primary motor cortex); SI, postcentral gyrus (primary somatosensory cortex); PCN, precuneus; PCC, posterior cingulate cortex; SubCalC, subcallosal cortex; SMG, supramarginal gyrus; SFG, superior frontal gyrus; SPL, superior parietal lobe; SMA, supplementary motor area; TP, temporal pole. *p < 0.05, **p < 0.001. All analyzed contrasts were corrected for multiple comparisons using the false discovery rate (FDR) at 0.05.

We found baseline rs-FC differences of the pain matrix between FM patients and HC [F(2,38) = 8.33, p = 0.03], with no interactions with behavioral data (pain catastrophizing, anxiety, and depression). FM patients showed higher rs-FC between the pain matrix (34 merged ROIs) and left precuneus (PCN), left SFG, right MidFG, right PaCiG, and left PCC; and lower rs-FC with right INS, when compared with HC. The analyses by seed revealed that FM patients display higher rs-FC of the following: left ACC, left AnG, right GP, left GP, left mPFC, right PCC, left PCC, left THA; and lower rs-FC of the following: right ACC, left AMYG, left AnG, left GP, left INS, right SMA (Fig. 3, Table 3). These results showed a disrupted rs-FC of the pain matrix in FM patients, when compared with HC. In our study, covariates did not show any significant influence in the main contrasts thus, rs-FC alterations in FM patients seem to be independent of age, years with FM diagnosis, as well as anxiety and depression symptoms. A total of 12 seeds from the original 34 were significantly altered when comparing FM vs HC, thus, only these seeds were included in the step-3 (rs-FC of MIA) analysis.

**Table 3:** Results of the baseline rs-FC contrast (FM vs HC).

Seed	Correlated Area	MNI Coordinates	β	T	p-FDR	Connectivity
Pain Matrix*	L PCN	−02, −60, 26	0.23	4.4	<0.001	Higher
	L SFG	−20, 40, 40	0.22	5.8	<0.001	Higher
	R MidFG	26, 26, 36	0.23	5.4	0.001	Higher
	R PaCiG	04, 52, 08	0.23	4.7	0.009	Higher
	L PCC	−06, −50, 18	0.22	4.6	0.05	Higher
	R INS	36, −20, −10	−0.21	−6.7	0.03	Lower
R ACC	R SI	60, −04, 36	−0.24	−5.2	0.006	Lower
L ACC	R STG	68, −16, 04	−0.22	−7.0	0.01	Higher
L AMYG	L CRBL	−26, −76, −30	−0.22	−5.7	0.04	Lower
L AnG	R PCN	06, −64, 40	0.31	6.2	<0.001	Higher
	L PaCiG	−04, 54, 18	0.29	7.5	<0.001	Higher
	R TP	58, −02, −26	0.28	7.4	<0.001	Higher
	L MTG	−56, −04, −26	0.31	6.4	<0.001	Higher
	L SubCalC	−06, 16, −20	0.25	6.4	<0.001	Higher
	L FP	−12, 40, 44	0.32	5.0	0.02	Higher
	R CRBL	10, −44, −48	0.23	6.1	0.02	Higher
	L INS	−30, 20, 10	−0.26	−7.6	0.02	Lower
	R SMG	64, −28, 26	−0.24	−4.6	0.02	Lower
	L MidFG	−46, 32, 26	−0.28	−4.7	0.02	Lower
	R INS	34, 20, 04	−0.24	−4.6	0.05	Lower
R GP	L IFG	−52, 16, 24	0.20	5.6	0.03	Higher
L GP	L SMG	−58, −40, 52	0.21	5.4	0.005	Higher
	R SMA	00, 02, 46	0.18	5.6	0.05	Higher
	R SPL	28, −46, 54	0.19	5.3	0.05	Higher
	L FP	−10, 44, 02	−0.22	−5.1	<0.001	Lower
	L PCN	−12, −60, 16	−0.19	−4.4	0.05	Lower
L INS	L PCN	−18, −58, 16	−0.22	−6.1	0.01	Lower
L mPFC	L AnG	−44, −58, 52	0.25	4.9	0.004	Higher
	R FP	20, 56, −06	0.29	5.4	0.01	Higher
R PCC	R PaCiG	02, 42, 26	0.26	5.9	<0.001	Higher
	L PCC	00, −38, 44	0.24	5.4	0.005	Higher
	R FP	42, 56, 14	0.22	7.1	0.02	Higher
	L AnG	−50, −60, 28	0.27	4.5	0.05	Higher
L PCC	R PCN	10, −56, 28	0.30	6.7	<0.001	Higher
	L SLOC	−40, −72, 32	0.30	6.8	<0.001	Higher
	R PaCiG	02, 44, 26	0.25	6.9	<0.001	Higher
	R SLOC	44, −54, 28	0.27	6.3	<0.001	Higher
R SMA	L SMA	−04, −16, 64	−0.29	−7.5	<0.001	Lower
	R TP	56, 16, −06	−0.27	−6.5	<0.001	Lower
	R M1	54, 00, 44	−0.24	−6.3	0.002	Lower
	L TP	−54, 18, −10	−0.23	−5.4	0.02	Lower
L THA	L PaCiG	−08, 34, 26	0.22	5.6	0.01	Higher
	PAG	00, −38, −48	0.19	6.0	0.02	Higher

FM, fibromyalgia patients; HC, healthy controls; BL, baseline condition; rs-FC, resting-state functional connectivity; β, effect size (positive effects represent higher connectivity; negative effects represent lower connectivity); T, T-value; p-FDR, p corrected false discovery rate; L, left; R, right. **Significant seeds**: Pain matrix (*34 seeds merged in an *a priori* mask); ACC, anterior cingulate cortex; AMYG, amygdala; AnG, angular gyrus; GP, globus pallidus; GP, globus pallidus; INS, insular cortex; mPFC, medial pre-frontal cortex; PCC, posterior cingulate cortex; PCC, posterior cingulate cortex; SMA, supplementary motor area; THA, thalamus. **Correlated areas**: AnG, angular gyrus; CRBL, cerebellum; FP, frontal pole; IFG, inferior frontal gyrus; INS, insular cortex; SLOC, superior lateral occipital cortex; MTG, medial temporal gyrus; MidFG, middle frontal gyrus; PaCiG, paracingular gyrus; PAG, periaqueductal gray matter; M1, precentral gyrus (primary motor cortex); SI, postcentral gyrus (primary somatosensory cortex); PCN, precuneus; PCC, posterior cingulate cortex; SubCalC, subcallosal cortex; SMG, supramarginal gyrus; SFG, superior frontal gyrus; SPL, superior parietal lobe; SMA, supplementary motor area; STG, superior temporal gyrus; TP, temporal pole. All analyzed contrasts were corrected for multiple comparisons using the false discovery rate (FDR) at 0.05.

### Resting state functional connectivity of music-induced analgesia

#### Main effects and interaction

**Figure 4:**
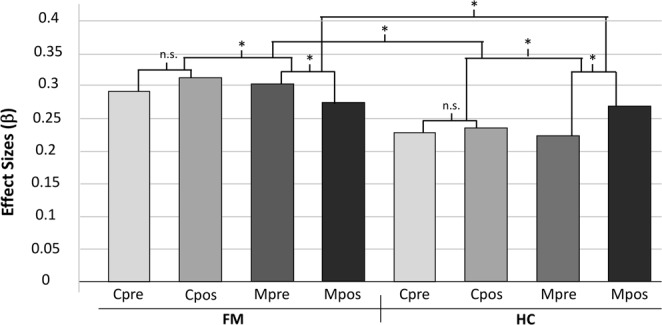
rs-FC of MIA in FM. The bars represent the effect size of each condition of rs-FC of the pain matrix. A 3-way interaction is obtained by main effect of group (FM vs HC), condition (music vs pink noise), and time (pre- vs post- test). FM patients rs-FC of the pain matrix is significantly different to that of HC. The experimental conditions have a different effect on rs-FC of the pain matrix in both groups and time points. The connectivity patterns before and after music, significantly differ in both groups, while pink noise has no significant effect. A 3-way interaction was found in the precuneus (see Table 4). FM, fibromyalgia; HC, healthy controls; rs-FC, resting-state functional connectivity. *significant effect; n.s., non-significant effect.

We found significant main effects of group (FM & HC), condition (music & pink noise) and time (pre- & post-test), and a 3-way interaction on rs-FC of the pain matrix (Fig. 4, Table 4). We conducted a GLM (α = 0.05), on a post hoc pain matrix that resulted from the baseline contrast (12 merged ROIs). In a 2 × 2 × 2 ANOVA, we modeled the different conditions (8 conditions; FM_Cpre, FM_Cpos, FM_Mpre, FM_Mpos, HC_Cpre, HC_Cpos, HC_Mpre and HC_Mpos) and conducted F-statistics to test main effects and interactions. The group main effect showed that FM display higher rs-FC of the pain matrix with left and right PCN, left INS, left PCC, and right AnG [F(8,152) = 4.47, p = <0.001]. Thus, FM patients and HC seem to process the experimental conditions in a different manner. The condition main effect showed that music has a significant effect on the pain matrix, with higher rs-FC of the pain matrix with right ACC [F(8,152) = 4.49, p = 0.02], when compared to pink noise. Thus, music and pink noise appear to display different connectivity patterns in the pain matrix of participants. The time main effect showed that the connectivity patterns were altered between time-points, possibly driven by rs-FC change in participants after listening to music [F(8,152) = 4.1, p = 0.02]. This effect was evidenced by higher rs-FC of the pain matrix with right AnG and right MidFG, and lower rs-FC of the pain matrix with right PaCiG and right PCN. Finally, we found a 3-way interaction between group, condition and time variables located in the right PCN [F(8,152) = 3.24, p = <0.001].

**Table 4:** Resting state functional connectivity of music-induced analgesia. Main effects and interaction.

Seed	Correlated Area	MNI Coordinates	β	p-FDR	Connectivity
***Group main effect*** (***FM vs HC***)***:*** [***F***(***8***,***152***) = ***4***.***47***, ***p*** = < ***0***.***001***]					
Pain matrix*	R PCN	02, −62, 34	0.30	<0.001	Higher
	L PCN	−04, −68, 28	0.35	0.004	Higher
	L INS	−30, 14, 02	0.32	0.004	Higher
	L PCC	−06, −54, 08	0.28	0.007	Higher
	R AnG	36, −68, 54	0.18	0.04	Higher
***Condition main effect*** (***Music vs Pink noise***)***:*** [***F***(***8***,***152***) = ***8***.***33***, ***p*** = ***0***.***02]***					
Pain matrix*	R ACC	02, 06, 42	0.35	0.02	Higher
***Time main effect*** (***Pre- vs Post-test***)***:*** [***F***(***8***,***152***) = ***4***.***1***, ***p*** = ***0***.***02***]					
Pain matrix*	R PaCiG	02, 48, 16	−0.17	0.02	Lower
	R AnG	30, −68, 34	0.19	0.02	Higher
	R MidFG	48, 24, 28	0.21	0.02	Higher
	R PCN	06, −76, 38	0.15	0.02	Lower
***3-way interaction*** (***Group*** × ***Condition*** × ***Time***)***:*** [***F***(***8***,***152***) = ***3***.***24***, ***p*** = <***0***.***001***]					
Pain matrix*	R PCN	02, −72, 34	0.30	<0.001	Higher

FM, fibromyalgia patients; HC, healthy controls; Mpre, pre-music; Mpos, post-music; β, effect size; p-FDR, p corrected false discovery rate; L, left; R, right. **Significant seed**: Pain matrix (*12 seeds that resulted from BL analysis, merged in a *post hoc* mask). **Correlated areas**: ACC, anterior cingulate cortex; AnG, angular gyrus; INS, insula; MidFG, middle frontal gyrus; PaCiG, paracingulate gyrus; PCN, precuneus; PCC, posterior cingulate cortex. All analyses were corrected for multiple comparisons using the false discovery rate (FDR) at 0.05.

### Post hoc contrasts

**Figure 5:**
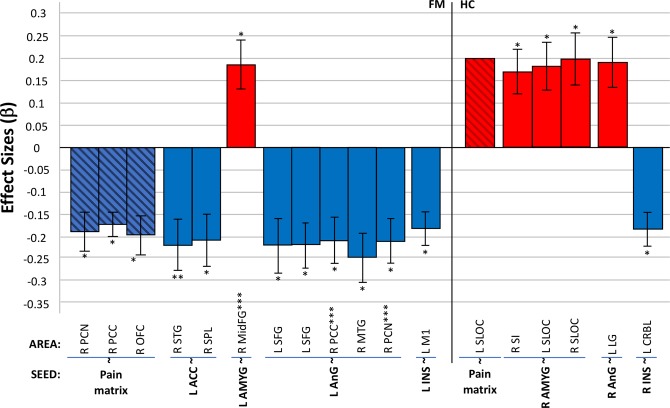
Mpre vs Mpos Contrast. Significant seed-to-voxel rs-FC of the pain matrix after listening to music for both groups (FM & HC); positive effects represent increased connectivity; negative effects represent decreased connectivity. FM, fibromyalgia; HC, healthy controls; rs-FC, resting state functional connectivity; L, left; R, right. Significant Seeds: Pain matrix (*12 seeds merged in a *post hoc* mask); ACC, anterior cingulate cortex; AMYG, amygdala; AnG, angular gyrus; INS, insular cortex. Correlated areas: CRBL, cerebellum; LG, lingual gyrus; MidFG, middle frontal gyrus; MTG, middle temporal gyrus; M1, precentral gyrus (primary motor cortex); OFC, orbitofrontal cortex; PCN, precuneus; PCC, posterior cingulate cortex; SI, postcentral gyrus (primary somatosensory cortex); SFG, superior frontal gyrus; SLOC, superior lateral occipital cortex; STG superior temporal gyrus; SPL superior parietal lobe. *p < 0.05, **p < 0.001, ***FC change correlated with pain scores (only ΔPI). All analyzed contrasts were corrected for multiple comparisons using the false discovery rate (FDR) at 0.05.

We conducted post hoc paired t-tests within each group to assess specific changes from pre to post-conditions. The Cpre vs Cpos contrast was not significant in either FM patients or HC, neither in the pain matrix nor by seed analyses. Thus, the control condition (pink noise) behaved as expected, and had no significant effect on rs-FC of the participants’ pain matrix. However, the Mpre vs Mpos contrast revealed that after listening to music, FM patients showed decreased connectivity of: (1) the pain matrix (12 merged ROIs) with right PCN, right PCC and right orbitofrontal cortex (OFC); (2) the left ACC with right posterior superior temporal gyrus (STG) and right superior parietal lobe (SPL); (3) the left AnG with right PCN, left superior frontal gyrus (SFG), right SFG, right PCC, and right posterior MTG; and (4) the left INS with left M1. They also showed increased connectivity of the left AMYG with right MidFG (Fig. 5, Table 5). Interestingly, we found that increased rs-FC of the pain matrix in the baseline condition, significantly showed opposite connectivity patterns after listening to music, namely a decrease of rs-FC. Additionally, increased rs-FC between left ACC and right STG (primary auditory cortex) in the baseline condition, significantly decreased after listening to music. Moreover, the increased rs-FC between left AnG and right PCN found in the baseline condition, also significantly decreased after listening to music. Thus, the connectivity changes after music may be interpreted as a “normalization” from the baseline disrupted rs-FC seen in FM patients when compared to HC.

**Table 5:** *Post hoc* paired t-test for Mpre vs Mpos rs-FC contrast analysis in FM patients and HC.

Seed	Correlated Area	MNI Coordinates	β	T	p-FDR	Connectivity
***Fibromyalgia patients***						
Pain matrix*	R PCN	01, −40, 46	−0.18	−6.2	0.02	Decreased
	R PCC	02, −18, 32	−0.17	−6.1	0.05	Decreased
	R OFC	48, 22, −14	−0.19	−7.6	0.02	Decreased
L ACC	R STG	68, −16, 04	−0.22	−7.1	<0.001	Decreased
	R SPL	18, −52, 72	−0.21	−6.2	0.04	Decreased
L AMYG	R MidFG	28, 30, 36	0.18	6.0	0.02	Increased
L AnG	R SFG	10, 16, 64	−0.22	−6.2	0.01	Decreased
	L SFG	−08, 18, 60	−0.22	−7.6	0.03	Decreased
	R PCC	02, −16, 48	−0.21	−7.1	0.05	Decreased
	R MTG	70, −16, −08	−0.25	−8.0	0.05	Decreased
	R PCN	10, −56, 28	−0.21	−7.0	0.05	Decreased
L INS	L M1	−18, −26, 56	−0.18	−8.7	0.03	Decreased
***Healthy controls***						
Pain matrix*	L SLOC	−48, −50, 40	0.20	4.5	0.03	Increased
R AMYG	R SI	56, −14, 32	0.17	5.4	0.02	Increased
	L SLOC	−20, −74, 34	0.18	7.1	0.03	Increased
	R SLOC	16, −80, 44	0.20	5.0	0.04	Increased
R AnG	L LG	−14, −56, −02	0.19	5.9	0.03	Increased
R INS	L CRBL	−38, −86, −24	−0.18	−6.4	0.03	Decreased

FM, fibromyalgia patients; HC, healthy controls; Mpre, pre-music; Mpos, post-music; rs-FC, resting-state functional connectivity; β, effect size; T, T-value; p-FDR, p corrected false discovery rate; L, left; R, right. **Significant seeds**: Pain matrix (*12 seeds that resulted from BL analysis, merged in a *post hoc* mask); ACC, anterior cingulate cortex; AMYG, amygdala; AnG, angular gyrus; INS, insular cortex. **Correlated areas**: CRBL, cerebellum; LG, lingual gyrus; MidFG, middle frontal gyrus; MTG, middle temporal gyrus; M1, precentral gyrus (primary motor cortex); OFC, orbitofrontal cortex; PCN, precuneus; PCC, posterior cingulate cortex; SI, postcentral gyrus (primary somatosensory cortex); SFG, superior frontal gyrus; SLOC, superior lateral occipital cortex; SPL superior parietal lobe; STG superior temporal gyrus. All analyzed contrasts were corrected for multiple comparisons using the false discovery rate (FDR) at 0.05.

The HC group also showed significant changes in rs-FC after listening to music. We found increased connectivity of: 1) pain matrix (12 merged ROIs) with left superior lateral occipital cortex (SLOC); 2) the right AMYG with right SI, left SLOC, and right SLOC; 3) the right AnG with left lingual gyrus (LG); and 4) the right INS with left CRBL (Fig. 5, Table 5). In other words, HC showed an overall increase of rs-FC after listening to music, that could be secondary to cognitive and emotional processes, as the pain-related regions are not exclusive to pain. Finally, we found no significant interactions with age, anxiety or depression symptoms in either of the groups.

### Correlation of resting state functional connectivity and music-induced analgesia

**Figure 6:**
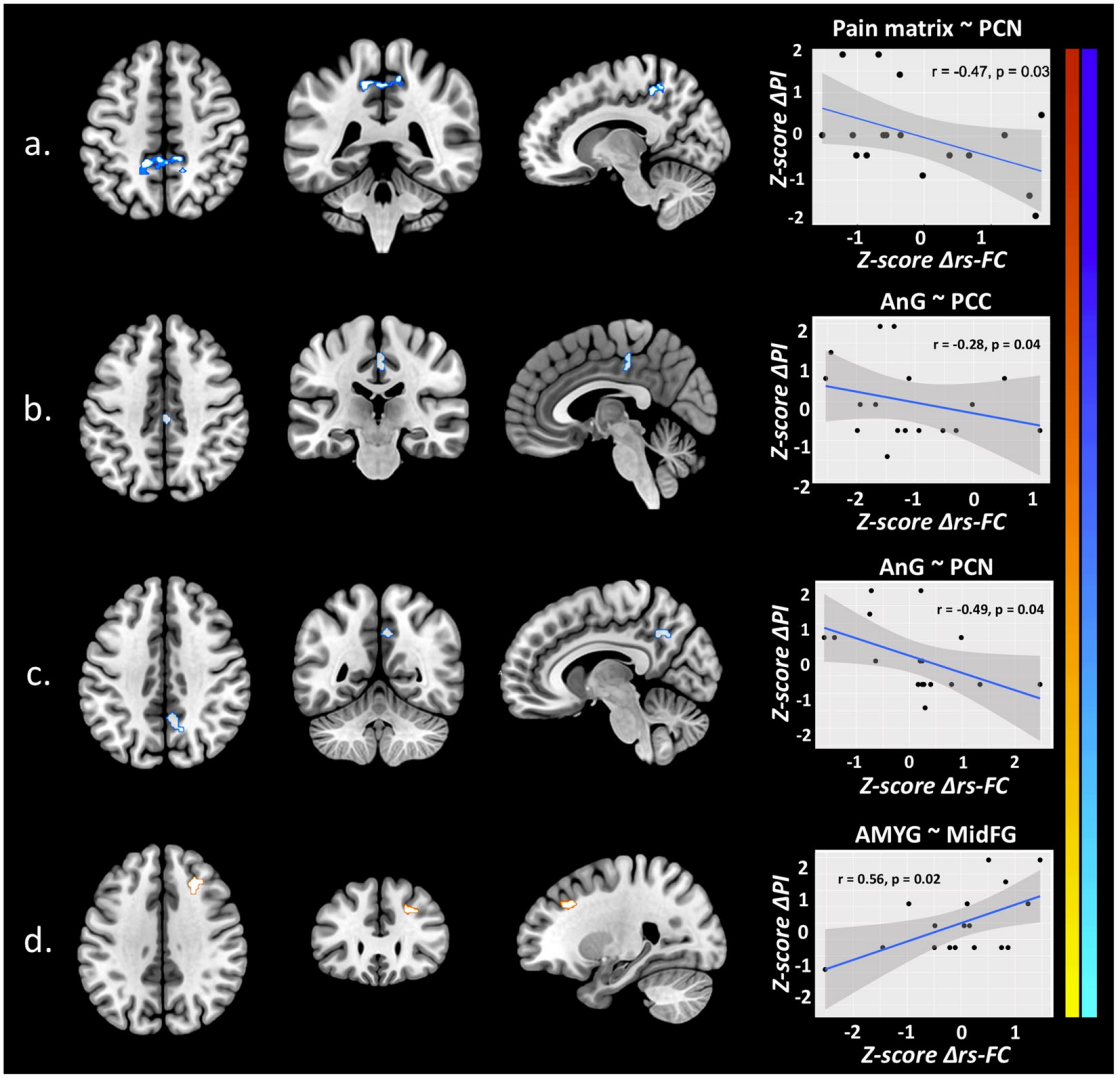
Music-Induced Analgesia correlates with rs-FC changes in FM patients. Scatterplot/regression line. Correlation between Mpre vs Mpos contrast (∆rs-FC) and ∆PI in FM patients. (**a**) Pain matrix ~ PCN; (**b**) AnG ~ PCC; (**c**) AnG ~ PCN; (**d**) AMYG ~ MidFG; FM, fibromyalgia patients; Mpre, before music; Mpos, after music; ∆PI, difference of pain intensity; ∆rs-FC, difference in Mpre vs Mpos rs-FC contrast; AMYG, amygdala; AnG, angular gyrus; PCC, posterior cingulate cortex; PCN, precuneus; MidFG, middle frontal gyrus; r, Pearson’s correlation coefficient.

We found significant correlations between pain scores and rs-FC of the pain matrix. First, we found a negative correlation between the decrease of pain intensity (ΔPI) and rs-FC decrease of the pain matrix (12 merged ROIs) with right PCN (r = −0.47, p = 0.03) after listening to music. Then, we also found results while testing correlation by seed, namely significant correlations between the change of pain intensity (ΔPI) and the change of in rs-FC in FM patients after listening to music (Δrs-FC). ΔPI was negatively correlated with the decrease of rs-FC between: 1) left AnG and right PCC (r = −0.28, p = 0.04), and 2) left AnG and right PCN (r = −0.49, p = 0.04), and it was positively correlated with the increase of rs-FC between left AMYG and right MidFG (r = 0.56, p = 0.02) (Fig. 6). In other words, the greater the analgesic effect the greater the decrease of rs-FC between AnG, PCC and PCN, and the greater the increase of rs-FC between AMYG and MidFG. ΔPU did not show any significant correlation with rs-FC of the pain matrix after listening to music.

## Discussion

In our study, we investigated the brain resting state functional connectivity (rs-FC) patterns related to music-induced analgesia in fibromyalgia (FM) patients. We found that the rs-FC of several brain regions from the pain matrix were already affected in FM patients, and that the rs-FC of those regions changed (reduced) after listening to music and correlated with analgesia reports.

### Disrupted resting state functional connectivity in fibromyalgia

Our results showed baseline rs-FC differences of the pain matrix between groups. FM patients showed higher rs-FC between the pain matrix (34 merged ROIs) and left precuneus (PCN), left SFG, right MidFG, right PaCiG, and left PCC; and lower rs-FC with right INS, when compared with HC. FM patients displayed significantly higher rs-FC on cingulate cortex (posterior and anterior) and mPFC, parietal cortex (AnG), and subcortical regions (GP and THA), than healthy controls (HC). The higher rs-FC of frontal and parietal regions with precuneus, may suggest a dynamic coupling of the default mode network (DMN) during pain perception, secondary to a focused attention on their condition^59^. FM patients may continuously engage autobiographical and self-awareness processes, commonly produced during rumination^60^. Our results seem to be consistent with previous neuroimaging studies demonstrating that these regions are activated during pain perception^61^ and are affected in FM^32^. Additionally, the higher rs-FC between the THA and the periaqueductal gray (PAG) may relate to the neuronal facilitation hypothesis of pain input into the central nervous system^5,38,62^, though functional connectivity does not convey directionality or the type of neuronal function involved (excitation or inhibition)^63–65^. Previous studies have found several brain regions such as the PAG, insula (INS), frontal pole (FP), amygdala (AMYG), and rostral ventral medulla (RVM), are involved in the descending pain modulatory system (DPMS)^6,66,67^. Finally, the higher rs-FC of basal ganglia (GP) with SMG and SMA may play a role in the integration of motor, emotional, autonomic and cognitive aspects of pain in FM, with an enhanced function of areas related to pain processing^35^.

We also found lower connectivity in FM patients on several regions, such as the ACC, AMYG, AnG, INS and SMA in FM patients. The lower rs-FC of the right SMA with M1, contralateral SMA and bilateral TP may explain a disrupted connectivity of motor areas with limbic and paralimbic regions. A proposed explanation for this is that the TP binds complex, highly processed perceptual inputs to visceral emotional responses^68^. FM patients seem to process emotion and pain in a different manner than the general population^69–71^, with relevant deficits in affective modulation measured by cardiac responses, heart rate variability, and neuroimaging^72^, suggesting alteration of emotional and attentional aspects of information processing in chronic pain^73^. Additionally, lower rs-FC between other emotion related regions such as AMYG and INS may support this hypothesis^30,37^. Finally, the lower rs-FC of ACC and AnG with INS and SI may relate to an altered somatosensory processing with limbic and pain related areas in FM patients^74–77^, which might be characterized by dissociation between sensory and affective components of pain-related information^73^. Somatic dysfunction in FM, including clinical pain, pain catastrophizing, autonomic dysfunction, and temporal summation, are closely related with the degree to which pain alters SI connectivity with affective pain processing regions^74^, and suggests that affective mood states can modulate central excitability thresholds in chronic pain states^69^.

### Music effects on pain and functional connectivity in fibromyalgia

We found that listening to music reduced pain in FM patients, and that this effect was related to significant reduction in rs-FC of pain matrix regions. Additionally, we found that the rs-FC of the pain matrix in healthy controls increased after listening to music. This suggests that rs-FC changes after listening to music affected each group differently, as hypothesized. Although our seeds for rs-FC were selected based on prior evidence related to pain perception^28,55–57^, these regions are not exclusively related to pain, i.e. INS^78,79^. Overall, we found that after listening to music, FM patients showed decreased rs-FC of the pain matrix with right PCN, right PCC and right OFC. After testing each seed independently, we found decreased rs-FC in left ACC, left AnG, and left INS seeds with mostly right regions, and increased in left amygdala with right MidFG.

#### The anterior cingulate cortex

The ACC has been described as a main hub in cognitive control, from reward processing and performance monitoring, to the execution of control and action selection^80^ and even suggested to be specific to pain processing^81^. There is evidence that the ACC is involved in processing the affective and unpleasant aspects of pain^28^. In our study, the decreased rs-FC of the ACC with the STG (primary auditory cortex) after listening to music may suggest an influence of music and sound processing in the modulation of pain that may not be explained solely by distraction, as the rs-fMRI was acquired after the music listening^82,83^. Music-evoked memories may play a role in the sustained distraction that prevails with the analgesic effect^84^. Noticeably, the increased rs-FC between ACC and STG in FM patients reported in the baseline condition, was significantly reduced after listening to music, thus, counteracting the altered rs-FC of this important pain-related area. Additionally, our study showed that the decreased rs-FC of ACC with SPL (somatosensory association) after listening to music may suggest disentanglement between areas closely related to pain at the cortical level, that may be related to the analgesic effect^85^. A possible mechanism may suggest a shift in the activity of the ACC from sensory processes to executive control activity by engaging in listening to preferred and pleasurable music. Thus, it seems that music has the ability to reduce the connectivity of the ACC with sensory areas, consequently reducing pain perception.

#### The default mode network

The DMN is large scale network with areas interacting among themselves in a highly correlated manner^86^. This set of areas appear to be more active when a person is not performing a task, but rather in a mind-wandering state^87,88^. Neuroimaging studies have evidenced increased activity while thinking about others or oneself, remembering the past and planning the future^89^.

Traditionally, the DMN is anticorrelated with the attention networks, and has been described to have three functional hubs: PCC/PCN, mPFC and AnG^88^. The PCC is an important hub in three networks: (1) the DMN, with increased activity during autobiographical memory^90^; (2) the dorsal attention network, with increased activity during visual attention and eye movement^91^; and (3) the frontoparietal control network, involved in executive motor control^92^. Thus, the PCC is specialized in shifting connectivity patterns. The PCN is an area of the DMN involved with episodic memory, visuospatial processing, memory-related imagery, reflections upon self, and aspects of consciousness^93–96^. It has been proposed to be a small-world network hub connecting parietal and prefrontal areas^97^. The mPFC is a frontal area involved in memory and decision making^98^. As part of the DMN, the mPFC is active while taking decisions about self, such as personal information, autobiographical memories, future goals and events, and decision making regarding our close companions (family)^88^. Additionally, studies have shown that the mPFC and OFC are involved in self-evaluation^99^, and the later has been related with emotion and reward in decision making. The AnG has been related to several brain functions including semantic processing, word reading and comprehension, number processing, memory retrieval, attention and spatial cognition, reasoning, and social cognitions^100,101^. It has been shown to be an important hub of the DMN, connecting perception, attention and spatial cognition during mental navigation at rest^86,89^. Although the AnG has not been traditionally related to pain, we decided to include it as a ROI based on two main considerations: 1) our previous study^40^ showed increased amplitude of the BOLD signal in AnG after listening to music, which correlated with analgesia reports, and 2) mental processes related to the DMN, such as autobiographic memory, awareness, and theory of mind, have been shown to be altered in FM^102,103^.

In the context of pain, neuroimaging studies have evidenced that various types of clinical chronic pain conditions are associated with rs-FC changes within the DMN^35,55,104^. The reorganization seem to linked to the connectivity between the mPFC and INS, and its dissociation from the posterior components of the DMN, which seem to disrupt the competitive inhibition between the DMN and the brain networks underlying attention^56,104^. Furthermore, FM patients have shown to display altered rs-FC not only of pain-related areas, but also altered function of the DMN^103^.

In our study, the pain matrix of FM patients showed a significant decrease of rs-FC with right PCN, right PCC and right OFC, after listening to music. After testing each seed independently, it seems that the decrease of rs-FC between AnG and SFG (premotor and SMA) after listening to music may suggest disengagement of areas related to pain and attention^89,101^. Interestingly, the decrease of rs-FC between AnG, PCC and PCN, may suggest a decrease in the activity of the DMN after listening to music, which may be related to the centralized analgesic effect in FM. Additionally, the decrease of rs-FC with OFC may suggest emotional and cognitive mechanisms such as reward while listening to preferred and pleasurable music. Noticeably, the increased rs-FC between these areas in FM patients reported in the baseline condition, was significantly reduced after listening to music, and significantly correlated with analgesia reports (higher ΔPI). Thus, a possible counteracting mechanism may occurred, that diminished the disruption in rs-FC of DMN-related areas in FM. Therefore, we may suggest a temporary disruption of autobiographical memory and self-awareness of the pain after listening to preferred music, e.g. disengaging the patient’s state of rumination.

#### The dorsolateral prefrontal cortex

The dlPFC is a functionally and structurally heterogenous region located in the MidFG^105^, implicated in cognitive^106^, affective^107^, decision-making^108^ and memory processing^109^. It is often associated with maintenance and regulation of top-down modulation, and driving appropriate behavioral responses^110,111^. The dlPFC is a key node of the extrinsic mode network^112^ and the cognitive control network^113^. In the context of pain, the dlPFC could modulate pain perception through these networks: (1) by controlling the regulation of cognitive networks through effective switching of the default mode network and extrinsic mode network; (2) by increasing the DPMS activity; and (3) by reducing emotional reactivity to pain through reward/fear circuitry^114^. The dlPFC has shown to be active in response to nociceptive stimuli in HC, and has shown different activation patterns between chronic pain patients and HC^114^. The dlPFC has also been implicated in pain suppression mechanisms, as seen in studies of placebo modulation of pain^115^, by integrating incoming nociceptive signals with the expectation of pain^116^. Furthermore, perceived control of pain has been associated with activity of the right dlPFC^117^, evidencing a role in the cognitive component of the pain experience. In our study, we found an increase of rs-FC between right MidFG and left AMYG after listening to music. It seems that after music listening, these two areas increase their connectivity and work synergistically to modulate pain perception via cognitive control. Noticeably, the increase of rs-FC between these areas after listening to music significantly correlated with analgesia reports (higher ΔPI).

#### The limbic areas

The structures and interacting areas of the limbic system have been involved in emotion, attention, learning, memory and motivation^118^. Our results show two important areas such as amygdala and insula with altered baseline rs-FC in FM patients compared to HC. Also, FM patients changed the connectivity patterns of these areas after listening to music. The AMYG is an integrative part of the limbic system, involved in many cognitive processes and largely considered as the most primordial and vital part of the limbic system. It has been shown that the AMYG encodes, stores, and retrieves episodic-autobiographical memories^119^. Moreover, the AMYG also seems to be an important region involved in emotional and attentional processes. Historically, this area has been thought to be linked to fear, allowing individuals to take a quick action in response to a threat^120^. However, new research has shown an underlying mechanism in which the AMYG helps an organism to define a stimulus and respond accordingly^121^. In the context of pain, the AMYG has also emerged as an important area for the emotional dimension of pain and pain modulation^122^. The AMYG has a strong network-level interaction with large-scale cognitive/affective cortical networks in chronic pain^123^. It seems that pain generates hyperactivity in the network of lateral, basolateral and central nuclei of the amygdala, which accounts for the emotional dimension of pain. The AMYG has shown strong connections with frontal areas, and altered function may result in declined function (e.g. cognitive and executive). Thus, decreased frontal feedback output to the AMYG allow the uncontrolled persistence of AMYG hyperactivity, and persistence of pain. Thus, effective AMYG-prefrontal connectivity may reflect successful emotion regulation processes^124^. In our study, the increase of rs-FC between left AMYG and right MidFG after music listening may be secondary to the association between auditory attention^125^, memory retrieval^126^, and positive emotions^127^, consistent with the use of a known, pleasant and emotionally positive music track. Moreover, both areas have been related to processes that involve adequate stimulus definition and appropriate behavioral responses^110,111,121^. Noticeably, the increase of rs-FC between these areas after listening to music significantly correlated with analgesia reports (higher ΔPI).

The insular cortex is an anatomical hub with large connection with cortical and subcortical regions serving sensory, motor, emotional and cognitive functions. In the context of pain, it seems that the INS mediates pain-related negative affect, and studies have shown insular activation when a person experiences pain or observes pain in others^128^. In the context of music, the INS cortex is involved in central auditory processing with efferent projections to the primary auditory, auditory association, and post-auditory cortices^129^. Previous studies have found changes in connectivity of the INS produced by music-listening and musical training^130,131^. In our study, we found a decrease of rs-FC between INS and primary motor cortex after listening to music, which strongly suggests an analgesic effect, as these regions are usually activated during pain perception^85^. Additionally, an increased activity in the INS secondary to pleasure feelings with music^132^, may shift the insula’s connectivity from a motor region to limbic regions.

#### Music-Induced analgesia in fibromyalgia

It is important to mention that FM patients show an altered baseline rs-FC^35^ that seems to be the result of the chronic pain and/or disease. Consequently, the dysfunction in the DPMS may induce a reorganization of the “pain-analgesia network”, and in order to modulate pain and produce an analgesic effect the system may be utilizing other circuits. We found that FM patients show increased rs-FC between left ACC and right STG (primary auditory cortex) in the baseline condition, which significantly decreased after listening to music. Similarly, the increased rs-FC between left AnG and right PCN in the baseline condition, also significantly decreased after listening to music (Figs 3 and 5). Noticeably, only AMYG-MidFG, AnG-PCC and AnG-PNC rs-FC was significantly correlated with pain intensity scores (Fig. 6). This suggests that the analgesic effect may not directly affect the main regions of the DPMS “gate” such as the PAG, or that we are purely measuring pain reappraisal processed in those regions. Larger studies with better measurements may provide further information about this. From our results, we can suggest cognitive and emotional mechanisms underlying music-induced analgesia. These mechanisms may be produced from brain processes such as distraction, pleasure, familiarity, memory evoked emotions, or a combination of them. It seems that the main feature of MIA in FM relies on shifting connectivity patterns from pain-related areas to executive and cognitive control areas.

### Music effects on functional connectivity of healthy controls

Listening to music also showed significant changes in rs-FC of the pain matrix in healthy controls^133–135^. However, as opposed to FM patients, healthy controls mostly showed increased rs-FC on right amygdala and angular gyrus seed regions with occipital areas. A right insula seed reduced its rs-FC with left cerebellum. As mentioned in the beginning of this discussion, the areas selected to build the pain matrix are not exclusive for pain processing, and are active during other cognitive processes. The emotional valence of music may play a role in connecting areas related to limbic, somatomotor, memory and visual imagery processes^136^. The intensity of pleasure experienced from music listening suggests a relation with dopamine reward system of the brain, and neural activity in surrounding limbic regions, indicative of emotional arousal^137–139^. Right amygdala showed an increased rs-FC with SI and SLOC in both hemispheres after listening to music, which are parietal areas of somatosensory functions and occipital areas involved in visual mental imagery^140^, and therefore, we suggest that music may engage visual imagery in healthy controls as seen in previous studies^141^. We found increased rs-FC of the AnG with the LG, possibly secondary to visual memory and visuo-limbic processes engaged after listening to music^142^. We also found decrease of rs-FC between right insula and left cerebellum, which may be caused by a focused attention on music, and a probable state of relaxation in healthy adults^143^.

### Limitations

The FM patients in this study were under different types of medication and had several comorbidities that we could not control, and which may affect baseline rs-FC. However, our main results show an analgesic effect of self-chosen, pleasant familiar music, related to changes in rs-FC. To account for any possible comorbidity confounds, we analyzed the data using depression and anxiety covariates, where we found no significant effect of such. It should be noted that the dosage of music intervention in this study may be limited (5 min), and the precise duration of the analgesic effect may be variable; though it seems 5 minutes was enough to elicit MIA. Our FM patients were not blinded to the music condition, which can produce a bias. Given the nature of music, blinding for participants is near impossible and thus a control such as white noise is used instead. In fact, our control condition (pink noise) behaved as expected, as we did not find any significant effect of it on pain perception or rs-FC analyses. Noticeably, the functional connectivity results comparing pre and post music, resulted in only 6 seeds with a change of rs-FC after music in FM patients. We believe that is only reasonable to correlate this neurophysiological data with behavioral data (pain reports pre-post music). We acknowledge how this might appear as double-dipping, but in fact, we are trying to validate subjective data (pain-self report) with objective data (rs-FC). Correlation may be spurious too, hence, we also performed FDR correction on those. We must be careful when interpreting functional connectivity analysis. This type of analysis has the limitation of not measuring directionality, therefore we cannot discuss if one of the areas is driving the change in connectivity pattern. Furthermore, this change in connectivity may be secondary to increase or decrease of activity, which in a more neurobiological point of view, we cannot discuss either if it is an excitatory or inhibitory activity. All we can say is that the pattern of BOLD activity is either correlated or anti-correlated between regions of interest, and infer a change in brain processes. Finally, it should be noted that our HC group did not experience pain, thus, a comparative measure to pain was not possible.

## Conclusions

Our results show that fibromyalgia patients experienced an analgesic effect after listening to music, and this effect correlated with mostly a reduction in resting state functional connectivity between pain related regions, and areas of the default mode network processing emotion, memory retrieval, and auditory attention. It seems that the main feature of the analgesic effect in fibromyalgia relies on shifting connectivity patterns from pain-related areas to executive and cognitive control areas. Hence, we suggest that music-induced analgesia in fibromyalgia is a top-down mechanism, probably originated by distraction, relaxation, positive emotion, or a combination of these mechanisms.

## Supplementary information

Supplementary Information

## Data Availability

Data in BIDS is freely available in OpenNeuro.org (10.18112/openneuro.ds001928.v1.1.0 or https://openneuro.org/datasets/ds001928/versions/1.1.0).
