# Supplementary material for: Functional connectivity of music-induced analgesia in fibromyalgia
Source: Sci Rep. 2019 Oct 29;9:15486. doi: 10.1038/s41598-019-51990-4 (PMC6820536; doi:10.1038/s41598-019-51990-4)
Supplement: Supplementary file 1 — Supplementary Information [file 41598_2019_51990_MOESM1_ESM.pdf]

Supplementary Information

Functional connectivity of music-induced analgesia in fibromyalgia.

Pando-Naude, Victor[1,2,5], Barrios, Fernando A.[1], Alcauter, Sarael[1], Pasaye, Erick H.[1], Vase, Lene[3,4], Brattico, Elvira[5], Vuust, Peter[5,6], Garza-Villarreal, Eduardo A.*[2,5]
*Corresponding Author:
Eduardo A. Garza-Villarreal
Laboratorio Nacional de Imagenología por Resonancia Magnética (LANIREM),
Laboratorio B-03, Instituto de Neurobiología,
Universidad Nacional Autónoma de México campus Juriquilla,
Boulervard Juriquilla 3001, Juriquilla, Querétaro, CP 76230, México
Phone: +52 (442) 238-1038
E-mail: egarza@comunidad.unam.mx

Supplementary Table 1 reports the center of MNI coordinates that were used to create the pain matrix in our study. The ROIs were obtained from peak coordinates of several pain studies, both experimental and clinical[1–6], and cross-referenced with brain atlases (Harvard-Oxford atlas FSLview; Juelich Histological Atlas FSLview), and Neurosynth[7] using "pain" and "chronic pain" as search terms, to ensure the precision of the obtained coordinates. Most of the seeds were derived from the study by Cifre et al. (2012)[5], given that it showed for the first time a disrupted brain functional connectivity in FM patients.

Supplementary Table 1. MNI Coordinates of Seeds' Center for the Pain Matrix.

	**Right Hemisphere**			**Left Hemisphere**		
**Seed**	x	y	z	x	y	z
**ACC**[ab]	1	8	30	-2	8	30
**AMYG**[b]	26	0	-22	-24	-2	-22
**AnG**[b]	54	-57	33	33	-48	-60
**BA41**[b]	50	-21	7	-52	-19	7
**CAU**[ab]	14	4	20	-12	14	8
**GP**[ba]	18	-2	-4	-12	0	2
**INS**[a]	36	6	6	-48	12	-2
**M1**[c]	10	-30	70	-10	-26	68
**mPFC**[bd]	2	46	-16	-2	46	-16
**PAG**[eb]	6	-32	-10	-6	-32	-10
**PCC**[ab]	12	-56	6	-12	-56	6
**PUT**[ab]	28	6	-2	-22	8	-4
**SI**[a]	52	-16	44	-48	-24	52
**SII**[a]	52	-20	16	-58	-24	14
**SMA**[ab]	12	2	68	-12	2	68
**STS**[eb]	60	-46	-4	-60	-48	-4
**THA**[fb]	20	-18	12	-10	-22	6

MNI, Montreal Neurological Institute; ACC, anterior cingulate cortex; AMYG, amygdala; AnG, angular gyrus; BA41, primary auditory cortex; CAU, caudate; GP, globus pallidus; INS, insular cortex; M1, primary motor cortex; mPFC, medial prefrontal cortex; PAG, periaqueductal gray matter; PCC, posterior cingulate cortex; PUT, putamen; SI, primary somatosensory cortex; SII, secondary somatosensory cortex; SMA, supplementary motor area; STS, superior temporal sulcus; THA, thalamus. a, Gracely et al., 2002; b, Harvard-Oxford atlas FSLview; c, Juelich Histological atlas FSLview; d, Baliki et al., 2008; e, Zaki et al., 2007; f, Burgmer et al., 2009.

Pando-Naude, Víctor
Garza-Villarreal, Eduardo A.

Supplementary Figure 1 shows the boxplots for the Mann-Whitney Rank test, measuring the effect of experimental conditions on pain intensity (PI) and pain unpleasantness (PU) in fibromyalgia (FM) patients. The figure shows that pink noise and music were significantly different in both ∆PI (W=60, p=0.002) and ∆PU (W=65.5, p=0.004). In other words, FM patients reported lower pain levels after listening to music, but not after listening to the pink noise. Pink noise produced a small increase of pain perception in FM patients (~1point VRS), which was non- significant in both PI and PU.

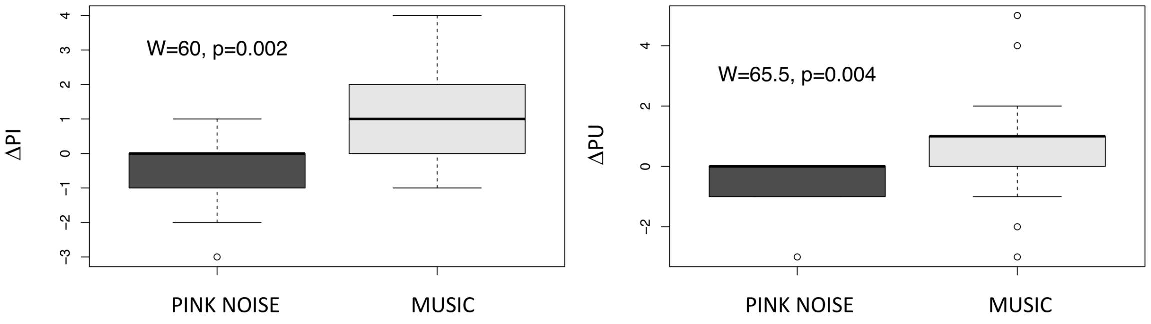

Supplementary Figure 1. Pain Intensity and Pain Unpleasantness Measures. Music vs Pink Noise; ∆PI, Difference in Pain intensity; ∆PU, Difference in Pain Unpleasantness; W, Mann-Whitney Rank test; p, statistical significance.

References

1. Gracely, R. H., Petzke, F., Wolf, J. M. & Clauw, D. J. Functional Magnetic Resonance Imaging Evidence of Augmented Pain Processing in Fibromyalgia. *ARTHRITIS Rheum.* **46,** 1333–1343 (2002).

2. Zaki, J., Ochsner, K. N., Hanelin, J., Wager, T. D. & Mackey, S. C. Different circuits for different pain: Patterns of functional connectivity reveal distinct networks for processing pain in self and others. *Soc Neurosci* **2,** 276–291 (2007).

3. Baliki, M. N., Geha, P. Y., Apkarian, A. V. & Chialvo, D. R. Beyond Feeling: Chronic Pain Hurts the Brain, Disrupting the Default-Mode Network Dynamics. (2008). doi:10.1523/JNEUROSCI.4123-07.2008

4. Burgmer, M. *et al.* Altered brain activity during pain processing in fibromyalgia. (2008). doi:10.1016/j.neuroimage.2008.09.008

5. Cifre, I. *et al.* Disrupted Functional Connectivity of the Pain Network in Fibromyalgia. *Psychosom. Med.* **74,** 55–62 (2012).

6. Garza-Villarreal, E. A. *et al.* Music reduces pain and increases resting state fMRI BOLD signal amplitude in the left angular gyrus in fibromyalgia patients. *Front. Psychol.* **6,** 1–11 (2015).

7. Yarkoni, T., Poldrack, R. A., Nichols, T. E., Van Essen, D. C. & Wager, T. D. Large-scale automated synthesis of human functional neuroimaging data. (2011). doi:10.1038/nMeth.1635
